# High serum superoxide dismutase activity improves radiation-related quality of life in patients with esophageal squamous cell carcinoma

**DOI:** 10.6061/clinics/2021/e2226

**Published:** 2021-04-16

**Authors:** Nannan Xue, Runze Zhou, Ming Deng, Yitong Li, Yong Hu, Liang Gao, Yunbo Zhang, Xiangyu Song, Junqi Liu, Ruitai Fan

**Affiliations:** I Department of Radiation Oncology, The First Affiliated Hospital of Zhengzhou University, Zhengzhou, Henan 453000, P.R. China.; II Department of Orthopedics, Renmin Hospital of Wuhan University, Wuhan, Hubei 430060, P.R. China.; III Department of Oncology, Hebei General Hospital, Shijiazhuang, Hebei 050000, P.R. China.; IV Department of Radiation Oncology, Hanzhong Central Hospital, Hanzhong, Shanxi 723000, P.R. China.; V Center of Experimental Orthopaedics, Saarland University Medical Center, Kirrberger Strasse, Homburg 66421, Germany.; VI Department of Oncology, Zibo Bashan Wanjie hospital, Zibo, Shandong 255000, P.R. China.; VII Department of Radiation Oncology, Linzhou people's hospital, Linzhou, Henan 456550, P.R. China.

**Keywords:** Esophageal Squamous Cell Carcinoma, Superoxide Dismutase, Radiation Pneumonitis, Leukopenia, Thrombocytopenia, Cohort Studies

## Abstract

**OBJECTIVES:**

Esophageal squamous cell carcinoma (ESCC) is one of the most common malignant tumors in China. Intensity-modulated radiation therapy and volume-modulated arc therapy have become the main treatments for esophageal carcinoma; however, side effects caused by radiotherapy greatly impact the quality of life in these patients. This study aimed to explore the impact of serum superoxide dismutase (SOD) levels on the prognosis of patients with ESCC undergoing radiotherapy.

**METHODS:**

Patients aged between 18 and 80 years with lower-middle ESCC who underwent radiotherapy were eligible for this assessment. Adverse events, responses, treatment outcomes, and overall survival (OS) were assessed. Between 2012 and 2014, 195 patients were enrolled, of which 65 were assigned to the low- and high-SOD groups based on their serum SOD values.

**RESULTS:**

The baseline characteristics were similar between the two groups, except for the T staging. Adverse events in the low-SOD group were significantly higher than those in the high-SOD group (radiation esophagitis, *p*=0.007; radiation pneumonitis, *p*=0.032; leukopenia, *p*=0.023; thrombocytopenia, *p*=0.037; anemia, *p*=0.041). There were no significant differences in response, treatment outcomes, or OS.

**CONCLUSION:**

In conclusion, high serum SOD activity improved post-radiotherapy quality of life but did not impact the prognosis of patients with ESCC. To the best of our knowledge, this study is the first to report that serum SOD activity is associated with radiation-induced toxicity and moderately increased radiotherapeutic response in patients with ESCC undergoing radiotherapy.

## INTRODUCTION

Esophageal squamous cell carcinoma (ESCC) is a common malignancy of the digestive tract that poses a serious threat to human health. In 2018, there were approximately 570,000 new cases worldwide, and 500,000 people died of esophageal cancer. More seriously, ESCC is one of the most common malignant tumors and is the fourth leading cause of cancer-related deaths in China, especially in Linxian, Henan province (1,2). Treatment modalities for ESCC include surgery, radiotherapy, and chemotherapy. Intensity-modulated radiation therapy and volume-modulated arc therapy, characterized by increased doses for tumor cells and greatly reduced doses for non-tumor cells, have become the primary radiotherapeutic treatment modalities for ESCC (3). Nevertheless, the side effects of radiotherapy, such as radiation esophagitis, radiation pneumonitis, and myelosuppression, are not only severely painful but may also affect treatment progress and patient compliance.

Reactive oxygen species (ROS), a natural by-product of normal metabolism, were first found in the process of adenosine triphosphate production in the mitochondria and plays an essential role in cell homeostasis and signaling (4). Peroxisomes and endoplasmic reticulum were also identified as critical ROS-producing organelles *in vivo* (5). As a second messenger of intracellular signaling, ROS mediate cell adhesion, immune response, apoptosis, proliferation, cell growth, and differentiation (6). X-rays above 33 eV destroy the hydroxide bonds in water to produce large quantities of ROS, which can inhibit enzyme activity and cause deoxyribonucleic acid (DNA) damage, including base damage, single-strand breaks, double-strand breaks, DNA-DNA crosslinks, and sugar damage (7-9). However, X-rays can damage non-tumor cells and cause various radiation-related side effects (10). Substances that are resistant to ROS oxidation include thioredoxin reductase, glutathione reductase, glutathione peroxidase, catalase, and superoxide dismutase (SOD), and they play a very critical role in cellular defense against oxidative damage (11).

In 1968, McCord and Fridovich found a protein in bovine red blood cells that can catalyze the dismutation of the superoxide (O_2_) radical into either hydrogen peroxide (H_2_O_2_) or ordinary molecular oxygen (O_2_) (12). They named this enzyme SOD. There are three major SOD families, including the copper/zinc-binding auxiliary group (Cu/Zn-SOD) (13), the manganese-containing metal prosthetic group (Mn-SOD), and the copper/zinc form. An increasing number of oncologists have focused on the ability of SOD to increase cellular radioresistance. Studies have shown that the overexpression of SOD could prevent mice from experiencing chronic ROS-induced damage from radiation or chemotherapy (14,15).

However, no clinical data have been published regarding the impact of SOD on patients with ESCC undergoing radiotherapy. In this retrospective study, we evaluated the role of SOD expression on adverse events, responses, treatment outcomes, and overall survival (OS) of patients undergoing radiotherapy.

## MATERIALS AND METHODS

### Patient selection

All patient data in this study were retrospectively obtained from the First Affiliated Hospital of Zhengzhou University, Hebei General Hospital, Linzhou People’s Hospital, and Hanzhong Central Hospital from February 2012 to February 2014 in China. Eligible patients were aged between 18 and 80 years, had been histologically and endoscopically confirmed to be newly diagnosed with ESCC, and had a Karnofsky Performance Score (KPS) of 70 or higher. The exclusion criteria were as follows: 1) cervical and upper thoracic esophageal cancer; 2) surgical treatment; 3) presence of distant metastasis; 4) presence of other organ dysfunction, such as insufficient pulmonary function, liver insufficiency, renal insufficiency, or myelosuppression (*i.e.* neutrophil count ≤1,500/mm^3^, platelet count ≤10×10^4^/mm^3^, hemoglobin ≤8.0 g/dL, creatinine ≤1.2 mg/dL); 5) history of radiotherapy or chemotherapy; 6) copper- or zinc-containing supplements taken before treatment, or other significant factors that interfere with the measurement if SOD including jaundice (bilirubin ≥200 μmol/L), lipids (chylomicrons ≥0.25%), and hemolysis; 7) only receiving chemotherapy without radiotherapy; 8) missing clinical information.

**Figure 1:**
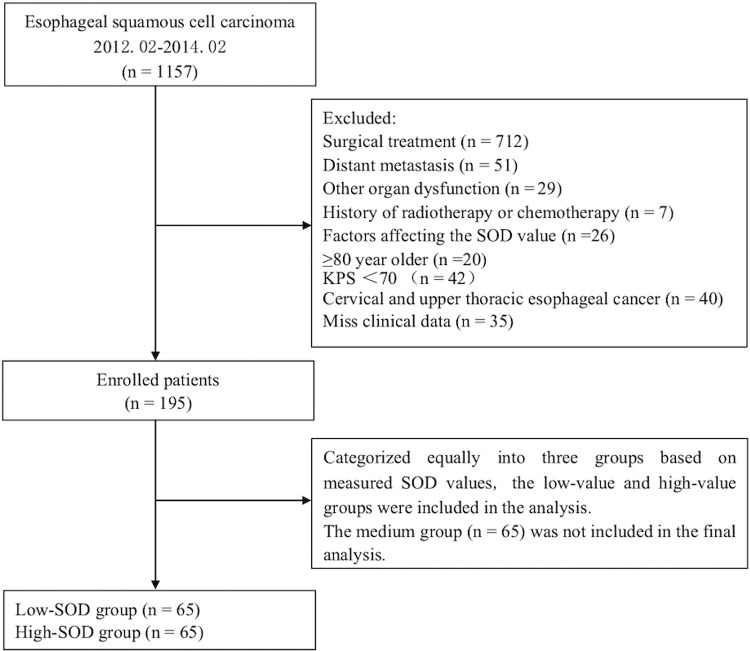
Flowchart depicting participant selection for this study.

A total of 195 patients who met the above criteria were recruited and divided equally into three groups based on their serum SOD values: low-, high- and median-SOD groups. Low- and high-SOD groups were used for the analysis (Figure 1). The study was approved by the Ethics Committee of the First Affiliated Hospital of Zhengzhou University.

### Procedures

All enrolled participants underwent an overall examination and assessment of their disease, including upper gastrointestinal endoscopy and tissue biopsy. Computed tomography and magnetic resonance imaging of the chest, upper abdomen, and neck were performed to determine the tumor stage according to the American Joint Committee on Cancer 8^th^ Edition Cancer Staging Form, and lymph node biopsy was performed for suspected lymph node metastasis. Sex, age, and occupation of the participants were documented, and KPS was evaluated, as were other clinical characteristics. All patients received standard concurrent chemoradiation, including RT+paclitaxel+carboplatin and RT+fluorouracil+cisplatin.

Blood samples from the median cubital vein were collected in sterile heparinized tubes and plasma was obtained by centrifuging the samples at 1,500g for 15 min after which they were immediately stored at -80°C for further analysis.

According to well-established methods (16), the SOD activity was determined using a Superoxide Dismutase Activity Assay Kit (cat. no. ab65354; Abcam, Cambridge, UK). Briefly, 15 μL of serum was used to evaluate the patients’ SOD levels. After allowing the reaction to proceed for 10 min, the absorbance of each sample was measured at a wavelength of 405 nm using a Siemens automatic biochemical analyzer (ADVIAI1800). The SOD activity levels were calculated using the manufacturer’s formula.

Toxicities were monitored weekly during treatment; the classification of toxicity was based on the Radiation Therapy Oncology Group toxicity criteria. Tumor responses to treatment were assessed according to the modified Response Evaluation Criteria in Solid Tumors.

### Statistical methods

OS was assessed using the Kaplan-Meier method, and comparisons between the low- and high-SOD groups were performed using the log-rank test. We compared the patient characteristics of the two groups using the chi-squared test for categorical variables and the Spearman rank correlation coefficient for grade data. Significance was defined as *p*<0.05. All statistical analyses were performed using the SPSS version 21.0.

## RESULTS

### Patient characteristics

The baseline characteristics of the groups are summarized in Table 1. There were no statistically significant differences in sex, age, N stage, clinical stage, and KPS between the two groups (*p>*0.05). However, 76.93% and 49.24% patients were stage T_3-4_ in the high- and low-SOD groups, respectively, and this difference was statistically significant (*p*=0.005).

### Toxicity

Non-hematologic adverse events included radiation esophagitis (82.31%) and radiation pneumonitis (50.00%) (Table 2). The incidence of radiation esophagitis by grade was 53.08% for grade 1 and 25.38% for grade 2. For grade 3 and grade 4, radiation esophagitis was rare (2.31% and 1.54%, respectively). The incidence of radiation esophagitis was extremely high in both the low- and high-SOD groups (83.08% and 81.54%, respectively), and this difference was statistically significant (*p=*0.007). A similar trend was observed for the incidence of radiation pneumonitis. The incidence of radiation pneumonitis in the low-SOD group was significantly higher than that in the high-SOD group (53.85% *versus* 46.15%, *p*=0.032), while two patients in the low-SOD group (3.08%) and one patient in the high-SOD group (1.54%) experienced grade 3 radiation pneumonitis. Hematologic adverse events included leukopenia (45.38%), thrombocytopenia (16.15%), and anemia (12.31%). The incidence of leukopenia was 50.77% in the low-SOD group and 40.00% in the high-SOD group. The most common severity of leukopenia was grade 3 in the low-SOD group (24.62%) and grade 1 in the high-SOD group (20.00%). The incidence of thrombocytopenia was 18.46% in the low-SOD group and 13.85% in the high-SOD group; seven patients (10.77%) had grade 3 thrombocytopenia in the low-SOD group and five patients (7.69%) had grade 1 in the high-SOD group. Ten patients in the low-SOD group (15.38%) and six patients in the high-SOD group (9.23%) experienced anemia; none experienced grade 3 or grade 4 anemia. The incidence of hematologic adverse events in the low-SOD group was also higher than that in the high-SOD group (*p*_Leukopenia_=0.023, *p*_Thrombocytopenia_=0.037, *p*_Anemia_=0.041).

### Responses

Short-term responses were assessed one month after treatment in both groups (Table 3). In the low-SOD group, 27 patients (41.54%) had a complete response, 21 patients (32.30%) had a partial response, 14 patients (21.54%) had stable disease, and three patients (4.62%) had progressive disease. In the high-SOD group, 29 patients (44.62%) had a complete response, 21 patients (32.30%) had a partial response, 10 patients (15.39%) had stable disease, and five patients (7.69%) had progressive disease. Long-term treatment outcomes were evaluated at one, three, and five years after treatment (Table 4). The 1-, 3-, and 5-year survival rates in the low-SOD group were 81%, 72%, and 66%, respectively, and those in the high-SOD group were 80.00%, 56.92%, and 53.30%, respectively, and there were no statistically significant differences between the two groups *(p=*0.694).

### Survival

**Figure 2:**
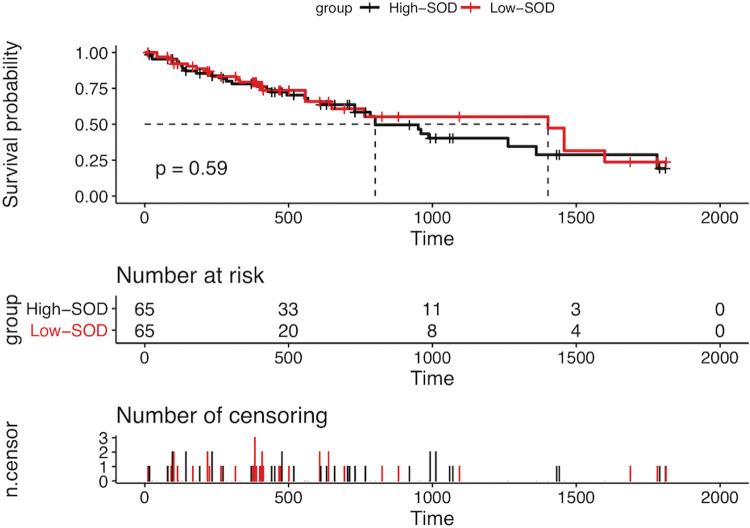
Kaplan-Meier curves of the high- and low-SOD groups. The 5-year OS rate in the low-SOD group was higher than the 5-year OS in the high-SOD group, but the difference was not statistically significant (stratified log-rank, *p*=0.585). SOD, serum superoxide dismutase; OS, overall survival.

The 5-year OS rates in the low- and high-SOD groups were 66.20% (43/65) and 52.3% (34/65), respectively. The median survival time in the low- and high-SOD groups was 801 days (95% confidence interval [CI]:583-1018 days) and 1,402 days (95% CI:776-2028 days). However, there was no statistically significant difference in survival time between the two groups (stratified log-rank test, *p*=0.585). The Kaplan-Meier curves are shown in Figure 2.

## DISCUSSION

Regarding the role of SOD in ESCC, previous studies have primarily focused on SOD expression levels in primary cancer and normal tissue and its mechanism for regulating the occurrence and development of esophageal cancer (17-20). It has been reported that SOD expression levels and activity are downregulated or absent in a series of cancers, including ESCC (19-22), breast cancer (23), prostate cancer (24), and lung adenocarcinoma (25), compared to normal tissues. Several studies have shown that overexpression of Mn-SOD contributes to a reduction in tumor invasiveness in ESCC and exerts a tumor-suppressive effect (26). However, studies exploring the impact of SOD on ESCC treated with radiotherapy have been limited to a few animal experiments; whether high SOD activity exerts a similar influence on patients with ESCC has not been reported in detail. To the best of our knowledge, this study is the first to report that serum SOD activity is associated with radiation-induced toxicity and moderately increased radiotherapeutic response in patients with ESCC undergoing radiotherapy.

Radiation-induced toxicities are considered critical dose-limiting factors in radiation therapy for esophageal cancer. The efficacy of SOD as a radioprotector has been demonstrated in a series of reports. Pingfan et al. and Hai-Xu et al. demonstrated that injecting SOD fusion protein into mice could attenuate radiation-induced lung injury (27,28). Vozenin-Brotons et al. and Delania et al. demonstrated that the intramuscular injection of liposomal Cu/Zn-SOD could reduce radiation-induced fibrosis in animal and human trials (29). Epperly et al. demonstrated that intraesophageal injection of clinical-grade liposomal Mn-SOD could decrease acute and chronic radiation-induced toxicities and improve radiation tolerance in mice (30). Our data are consistent with those of these studies. In patients with esophageal cancer undergoing treatment, we found that the incidence of radiation-induced toxicities, including radiation esophagitis, radiation pneumonitis, leukopenia, thrombocytopenia, and anemia, in the high-SOD group was significantly lower than that in the low-SOD group, indicating that high SOD activity could lead to improved radiotherapy tolerability compared to the low SOD activity. Yang et al. demonstrated that Mn-SOD notably decreased plasma concentrations of radiation-induced proinflammatory cytokines, including IL-1β, IL-6, and TNF-α, and boosted the production of the anti-inflammatory cytokine, IL-10 (27). Therefore, we speculate that high SOD activity not only provides an antioxidant effect but also strikingly enhances anti-inflammatory effects, which may explain why SOD can decrease the incidence of radiation-induced toxicities.

We further performed survival and response analyses. The Kaplan-Meier analysis for OS showed that, for approximately 800 days after treatment, the OS of the high-SOD group was generally consistent with that of the low-SOD group. After 800 days, the survival curves separated, and the high-SOD group outperformed the low-SOD group; at 1,600 days, the survival curves crossed, and the OS outcomes of the two groups remained similar. We speculate that this is because X-rays and γ-rays, as well as heavy particle radiation such as protons and neutrons, mediate the radiation-induced killing of cancer cells by augmenting oxidative stress (31). However, the suppression of ROS by SOD may have occurred in normal and tumor cells, leading to decreased tumor cell death in the high-SOD group. Nonetheless, the Kaplan-Meier curves showed that there was no significant difference between the groups, suggesting that any such suppression in tumor cells had minimal impact on the prognosis trends.

There are several potential limitations of the present study. First, all patient data in this study were enrolled between 2012 and 2014, during which standard treatment methods were not uniform. Consequently, the outcome of patients likely differed owing to differences in anti-tumor drugs and/or dosages. Second, this was a retrospective study with a limited sample size, and the accuracy of the results is subject to selection and recall biases, primarily due to the applied eligibility criteria. Future studies should focus on prospective clinical studies to confirm our results.

## CONCLUSION

This retrospective study demonstrated that high serum SOD activity might improve post-radiotherapy quality of life in patients with ESCC but does not significantly contribute to prolonged patient survival when compared to patients with low serum SOD activity. These results require further confirmation.

## AUTHOR CONTRIBUTIONS

Xue N wrote the manuscript and performed the data analysis. Hu Y, Song X and Li Y completed the clinical data collection. Zhou R revised the manuscript and collected data. Deng M and Gao L carried out data quality control. Zhang Y created the figure drawings. Liu J and Fan R designed the study.

## Figures and Tables

**Table 1:** Baseline patient characteristics.

	Low-SOD (n=65)	High-SOD (n=65)	Total (n=130)	*p-*value
Sex				0.594
Male	40 (61.54%)	36 (55.38%)	76 (58.46%)	
Female	25 (38.46%)	29 (44.62%)	54 (41.54%)	
Age				0.16
>65	27 (41.54%)	36 (55.38%)	63 (48.46%)	
≤65	38 (58.46%)	29 (44.62%)	67 (51.54%)	
T stage				0.005
T1	5 (7.69%)	2 (3.07%)	7 (5.38%)	
T2	28 (43.07%)	13 (20.00%)	41 (31.54%)	
T3	14 (21.55%)	23 (35.38%)	37 (28.46%)	
T4	18 (27.69%)	27 (41.55%)	45 (34.62%)	
N stage				0.659
N0	35 (53.85%)	30 (46.15%)	65 (50.00%)	
N1	12 (18.46%)	19 (29.23%)	31 (23.85%)	
N2	18 (27.69%)	16 (24.62%)	34 (26.15%)	
Clinical stage				0.513
1	8 (12.30%)	4 (6.15%)	12 (9.23%)	
2	34 (52.31%)	36 (55.38%)	70 (53.85%)	
3	23 (35.38%)	25 (38.46%)	48 (36.92%)	
KPS				0.554
70	7 (10.77%)	9 (13.85%)	16 (12.31%)	
80	15 (23.08%)	20 (30.77%)	35 (26.92%)	
90	19 (29.23%)	13 (20.00%)	32 (24.62%)	
100	24 (36.92%)	23 (35.38%)	47 (36.15%)	

Abbreviations: SOD, superoxide dismutase; KPS, Karnofsky Performance Score.

**Table 2:** Toxicity of patients in the low- and high-Superoxide dismutase groups.

	Low-SOD (n=65)	High-SOD (n=65)	Total (n=130)	*p-*value
Radiation esophagitis				0.007
1	28 (43.08%)	41 (63.08%)	69 (53.08%)	
2	23 (35.38%)	10 (15.38%)	33 (25.38%)	
3	2 (3.08%)	1 (1.54%)	3 (2.31%)	
4	1 (1.54%)	1 (1.54%)	2 (1.54%)	
All grades	54 (83.08%)	53 (81.54%)	107 (82.31%)	
Radiation pneumonitis				0.032
1	15 (23.08%)	21 (32.31%)	36 (27.69%)	
2	18 (27.69%)	8 (12.31%)	26 (20.00%)	
3	2 (3.08%)	1 (1.53%)	3 (2.31%)	
All grades	35 (53.85%)	30 (46.15%)	65 (50.00%)	
Leukopenia				0.023
1	8 (12.31%)	13 (20.00%)	21 (16.15%)	
2	7 (10.77%)	7 (10.77%)	14 (10.77%)	
3	16 (24.62%)	4 (6.15%)	20 (15.38%)	
4	2 (3.08%)	2 (3.08%)	4 (3.08%)	
All grades	33 (50.77%)	26 (40.00%)	59 (45.38%)	
Thrombocytopenia				0.037
1	1 (1.54%)	5 (7.69%)	6 (4.61%)	
2	3 (4.61%)	2 (3.08%)	5 (3.85%)	
3	7 (10.77%)	1 (1.54%)	8 (6.15%)	
4	1 (1.54%)	1 (1.54%)	2 (1.54%)	
All grades	12 (18.46%)	9 (13.85%)	21 (16.15%)	
Anemia				0.041
1	3 (4.61%)	5 (7.69%)	8 (6.15%)	
2	7 (10.77%)	1 (1.54%)	8 (6.15%)	
3	0	0	0	
4	0	0	0	
All grades	10 (15.38%)	6 (9.23%)	16 (12.30%)	

Abbreviations: SOD, superoxide dismutase.

**Table 3:** Tumor response rates in the high- and low-superoxide dismutase groups.

	High-SOD (n=65)	Low-SOD (n=65)	*p-*value
Complete response	29 (44.62%)	27 (41.54%)	0.758
Partial response	21 (32.30%)	21 (32.30%)	
Stable disease	10 (15.39%)	14 (21.54%)	
Progressive disease	5 (7.69%)	3 (4.62%)	

Abbreviations: SOD, superoxide dismutase.

**Table 4:** Survival rates of patients in the high- and low-superoxide dismutase groups.

	High-SOD (n=65)	Low-SOD (n=65)	*p-*value
1-year survival rate	52 (80.00%)	53 (81.50%)	0.694
3-year survival rate	37 (56.92%)	47 (72.3%)	
5-year survival rate	34 (53.30%)	43 (66.20%)	

Abbreviations: SOD, superoxide dismutase.
